# New York

**Published:** 1895-06

**Authors:** 


NEW YORK.

The Horseshoers' Bill in the New York Legislature has passed both the Senate and House, and now awaits the Governor's action. One of the most ardent workers for the success of this measure was Veterinarian S. K. Johnson, of New York City, in whom the master-shoers found a warm friend and supporter.

